# Tri-State Medical Society—Alabama, Georgia and Tennessee

**Published:** 1893-11

**Authors:** 


### TRI-STATES MEDICAL SOCIETY OF ALABAMA, GEORGIA AND TENNESSEE.

It was our pleasure to attend the fifth annual session of the Tri-State Medical Society which was held in Chattanooga, Tenn. Oct. 17th, 18th and 19th.

The attendance, while not as large as on some previous meetings, was satisfactory. The quality of the papers were above the average and provoked interesting discussions.

The success of the meeting is largely due to Dr. Douglass, whose indefatiguable work in this direction deserves the thanks of all the members.

Dr. Douglass' address, entitled "The Responsibilities of the Abdominal Surgeon," was exceedingly interesting, and the points brought out cannot fail to be of much value to the gynecologist and surgeon.

The *Tri-State Bulletin,* an advertising sheet, issued each morning of the meeting, was a convenient method of placing before the members the program and discussions of the day previous. We do not think, however, that such short reports of each paper and the discussions which followed were of sufficient scope to bring out the real merits of the papers.

A committee was appointed to revise the constitution.

The society decided that the next meeting should be held in Atlanta, Ga., the third Tuesday in October, 1894.

The following officers were elected:

Presideni—J. B. S. Holmes, Atlanta, Ga.

Vice-Presidents—James A, Goggans, Alexander City, Ala.; Dan. H. Howell, Atlanta, Ga.; T. Hilliard Wood, Nashville, Tenn.

Councillors—W. E. B. Davis, Alabama; G. W. Mills, Georgia; J. B. Murfree, Tennessee.

Secretary—Frank Trester Smith, Chattanooga, Tenn.

Treasurer—W. C. Townes, Chattanooga, Tenn.

Recorder—W. L. Gahagan, Chattauooga, Tenn.

